# Distinct contributions of hyperglycemia and high-fat feeding in metabolic syndrome-induced neuroinflammation

**DOI:** 10.1186/s12974-018-1329-8

**Published:** 2018-10-22

**Authors:** Brooke J. Wanrooy, Kathryn Prame Kumar, Shu Wen Wen, Cheng Xue Qin, Rebecca H. Ritchie, Connie H. Y. Wong

**Affiliations:** 1 Centre for Inflammatory Diseases, Department of Medicine, School of Clinical Sciences at Monash Health, Monash Medical Centre, Monash University, Clayton, VIC 3168 Australia; 2 Baker Heart and Diabetes Institute, Melbourne, Australia; 3 Department of Diabetes, Monash University, Melbourne, Australia

**Keywords:** Neuroinflammation, High-fat feeding, Microglia, Astrocytes, Hippocampus, Streptozotocin, Hyperglycemia

## Abstract

**Background:**

High-fat feeding and hyperglycemia, key risk factors for the development of metabolic syndrome (MetS), are emerging to associate with increased risk of developing dementia and cognitive decline. Despite this, clinical and experimental studies have yet to elucidate the specific contributions of either high-fat feeding or hyperglycemia to potential neuroinflammatory components. In this study, we delineate these individual components of MetS in the development of neuroinflammation.

**Methods:**

Male C57Bl/6 J adult mice were treated with either citrate vehicle (CIT) or streptozotocin (STZ; 55 mg/kg) 3, 5 and 7 days before commencement of either a normal or high-fat diet for 9 or 18 weeks. By creating separate models of high-fat feeding, STZ-induced hyperglycemia, as well as in combination, we were able to delineate the specific effects of a high-fat diet and hyperglycemia on the brain. Throughout the feeding regime, we measured the animals’ body weight and fasting blood glucose levels. At the experimental endpoint, we assessed plasma levels of insulin, glycated haemoglobin and performed glucose tolerance testing. In addition, we examined the effect of high fat-feeding and hyperglycemia on the levels of systemic inflammatory cytokines, gliosis in the hippocampus and immune infiltration in cerebral hemispheric tissue. Furthermore, we used intravital multiphoton microscopy to assess leukocyte-endothelial cell interactions in the cerebral vasculature of mice in vivo.

**Results:**

We showed that acute hyperglycemia induces regional-specific effects on the brain by elevating microglial numbers and promotes astrocytosis in the hippocampus. In addition, we demonstrated that chronic hyperglycemia supported the recruitment of peripheral GR1^+^ granulocytes to the cerebral microvasculature in vivo. Moreover, we provided evidence that these changes were independent of the systemic inflammation associated with high-fat feeding.

**Conclusions:**

Hyperglycemia alone preferentially induces microglial numbers and astrocytosis in the hippocampus and is associated with the peripheral recruitment of leukocytes to the cerebrovasculature, but not systemic inflammation. High-fat feeding alone, and in combination with hyperglycemia, increases the systemic pro-inflammatory cytokine milieu but does not result in brain-specific immune gliosis. These results shed light on the specific contributions of high-fat feeding and hyperglycemia as key factors of MetS in the development of neuroinflammation.

**Electronic supplementary material:**

The online version of this article (10.1186/s12974-018-1329-8) contains supplementary material, which is available to authorized users.

## Background

The prevalence of metabolic syndrome (MetS) in adults is increasing worldwide [1], largely due to several factors such as ageing of the population, increased life expectancy and chronic overnutrition and physical inactivity [2]. MetS refers to a cluster of inter-connected metabolic abnormalities involving glucose metabolism (diabetes milletus), lipid metabolism (hypercholesterolaemia and dyslipidaemia), elevated blood pressure and central obesity. In particular, obesity is emerging as a major health concern owing to its key role in MetS and being a well-recognised risk factor for the development of type 2 diabetes (T2D) and related cardiovascular diseases (CVDs) [3]. Recently, interdisciplinary research in neuroscience and immunology has linked overnutrition to neuroinflammation, particularly in the hypothalamus and hippocampus [4]. A growing body of evidence suggests that a complex interplay exists between accelerated adiposity, hyperglycemia and cognitive decline [5]. Indeed, clinical studies demonstrate that patients with T2D are more susceptible to symptoms of neurological impairment, but how T2D may induce neurological deficits remains undetermined.

The recently identified systemic and chronic inflammatory component of MetS is a primary candidate to confer the increased risk of cognitive decline in both obese and hyperglycemic individuals, perhaps by priming the specialised resident macrophage population (microglia) towards an inflammatory state to establish a form of low-grade neuroinflammation [6]. Chronic hyperglycemia in its own right is known to have deleterious effects on the brain. Meta-analyses of non-obese hyperglycemic type 1 diabetes patients show mild declines in mental speed and flexibility compared to non-diabetic individuals [7]. Moreover, increased blood-brain barrier permeability, oxidative damage and neuronal apoptosis are all associated with hyperglycemia in experimental animal models of hyperglycemia [8–10]. These conditions are seen similarly in conjunction with obesity, where negative relationships exist between body mass index (BMI) and neuronal viability, myelin integrity and grey matter volumes of the hippocampus in middle-aged adults [11, 12] However, both clinical and experimental studies have yet to elucidate the specific contributions of either obesity or hyperglycemia to its implicated neuroinflammatory component. Foundational animal studies have primarily investigated the contributions of obesity and hyperglycemia in mice deficient in either the leptin (*ob/ob*) or leptin receptor (*db/db*) as a result of genetic mutations. While beneficial, findings in these models have often proven difficult to translate to, and do not accurately reflect, the disease aetiology of obesity and diabetes in humans [13]. Furthermore, these models do not allow for the analysis of individual components of MetS in the development of neuroinflammation.

The present study was designed to dissect the specific contributions of high-fat feeding and hyperglycemia to neuroinflammation. We hypothesise that the systemic inflammatory component as a result of high-fat feeding is required to initially activate the brain’s immune system, which may be worsened by progressive hyperglycemia. To test our hypothesis, we assessed changes to systemic inflammatory cytokines, microglia numbers and astrocytosis in the hippocampus, and immune cell infiltrate in the brains of high-fat diet (HFD)-fed and streptozotocin (STZ)-treated C57BL/6 mice over 9 and 18 weeks. We also used intravital multiphoton microscopy to assess leukocyte-endothelial cell interactions in the cerebral vasculature of these mice in vivo.

## Methods

### Mice

Five to 6-week-old adult male C57Bl/6 J mice were obtained from the Monash Animal Research Platform and were housed in specific pathogen-free (SPF) conditions with access to food and water ad libitum (Monash Medical Centre Animal Facility, Clayton, VIC, Australia). Mice were housed 2–5 per cage in temperature-controlled rooms (22 °C) under a standard 12 h light-dark cycle. Prior to the start of experiments, mice were acclimatised for a minimum of 7 days before use. Mice were monitored every 3–4 days for specific health monitoring parameters including body weight, alertness, activity, coat health, dehydration, and gait. All procedures were approved by the Monash University Animal Ethics Committee under regulations that comply with the National Institute of Health guidelines for the care and use of laboratory animals.

### Induction of hyperglycemia and high-fat feeding

After acclimatisation, all mice were randomly assigned treatment groups. Mice for use in the control group underwent three intraperitoneal (IP) injections of citrate vehicle (CIT, 0.01 M, pH 4.5) 3, 5 and 7 days prior to commencement of control diet (CON; SF09-091, 16% kilojoules from lipids, Speciality Feeds, WA) for 9 or 18 weeks. These mice were designated with the group name “CIT + CON”. To develop a high-fat feeding model, a separate group of mice was treated identically with citrate vehicle and were placed on a high-fat diet (HFD; SF16-104, 44% kilojoules from lipids, Specialty Feeds, WA) for 9 or 18 weeks and designated “CIT + HFD”. To develop a mild hyperglycemia-only model, mice were treated with three separate IP injections of low-dose streptozotocin within 5 min of preparation (STZ; 55 mg/kg in 0.01 M CIT) 3, 5 and 7 days prior to the commencement of control diet for 9 or 18 weeks. These mice were designated with the group name “STZ + CON”. To develop a combination model of both hyperglycemia and high-fat feeding, mice were treated with three separate IP injections of STZ (55 mg/kg in 0.01 M CIT) 3, 5 and 7 days prior to the commencement of a HFD for 9 or 18 weeks. These mice were designated the group name “STZ + HFD”. Low-dose streptozotocin (STZ) treatment was selected as it causes the targeted destruction of the insulin-producing β cells of the pancreas to produce mild hyperglycemia [14].

### Glucose and insulin measurements

Fasting blood glucose was monitored fortnightly in all mice as a health monitoring parameter using a hand-held glucometer (Accu-Chek Performa, Roche) from whole blood collected via saphenous tail vein bleeds. Mice were fasted for 3 h prior to blood collection. Two weeks before end-point, a glucose tolerance test was performed. Baseline fasting blood glucose levels were obtained and mice were then injected IP with 0.8 g/kg glucose solution. Further measurements were taken at 15, 30, 45, 60, 90 and 120 min post-injection via tail vein bleeds. In addition, glycated haemoglobin (HbA1c) was assessed at the experimental endpoint. Mice were anaesthetised with isofluorane prior to transcardiac puncture with a heparinised 26G needle (100 I.U/mL). A small aliquot of whole blood (20 μL) was used for HbA1c analysis, via the Cobas B 101 (Roche Diagnostics). Plasma was collected from the remaining blood and stored at − 80 °C until required. Insulin levels were evaluated from plasma using a mouse ultrasensitive insulin ELISA kit (Alpco, Beijing, China) in single replicates according to the manufacturer’s instructions.

### Flow cytometry of brain immune cell isolates

The left cerebral hemisphere was cut into small pieces and digested in 500 μL collagenase digestion buffer at 37 °C for 30 min (collagenase XI (125 U/mL), hyaluronidase (60 U/mL) and collagenase type I-S (450 U/mL) in DPBS (Ca^2+^/Mg^2+^ supplemented)). Samples were kept on ice and passed through a 70 μm nylon cell strainer. Single-cell suspensions were subjected to 30% isotonic Percoll, underlaid with 70% Percoll and centrifuged for 20 min at 600*g* at room temperature. The interphase containing the mononuclear cells was collected, washed and stained with eFluor 506-conjugated anti-mouse CD45 (30-F11, eBioscience), FITC-conjugated anti-mouse CD3 (145-2CC, eBioscience), eFluor 450-conjugated anti-mouse B220 (RA3-6B2, eBioscience), PE-Cy7-conjugated anti-mouse CD11b (M1/70, eBioscience) and PE-conjugated anti-mouse CD68 (FA/11, eBioscience) with Fc-receptor blocker (1:100, 2.4G2, BD Pharmingen) for 15 min on ice in the dark. After staining, the cells were washed, resuspended with counting bead mixture (50 / μL) and 7AAD live-dead viability stain (1:50, 420404, Biolegend) in FACS buffer. Samples were run using a flow cytometer (BD FACSCanto II) and analysed using FlowJo (v10.3.0). Cell numbers were normalised to the left cerebral hemisphere weight of each mouse.

### Immunofluorescence

Mice were deeply anaesthetised with a cocktail of ketamine (150 mg/kg) and xylazine (10 mg/kg) prior to cardiac perfusion. An 18G needle was inserted into the left ventricle to perfuse ice-cold PBS and subsequent 4% paraformaldehyde (PFA; in PBS, pH 7.4). After perfusion, the brain was left to fix in PFA for 1 h and cryoprotected in 20% sucrose overnight. Brains were frozen in isopentane for cutting using a cryostat. Tissue anterior to the hippocampus was discarded until the defining bowtie-like structures of Cornu Ammonis (CA1) could be visually identified. Sections were cut at 10 μm and left at room temperature for 30 min prior to storage at − 80 °C until required. Upon use, sections were acclimatised to room temperature prior to rehydration with Tris-buffered saline containing 0.1% Tween 20 (TBST, pH 7.6), thrice, for 5 min. Sections were encircled with hydrophobic ink to maintain a staining well. Sections were permeabilised in 0.5% Triton X-100 (in 1x TBST, pH 7.6) at room temperature for 15 min in a humidifying chamber. Sections were then incubated for 48 h at 4 °C with either APC-conjugated anti-mouse Iba1 (1:1000, NCNP24, Wako Chemicals) to label microglia, anti-mouse GFAP (1:250, 1B4, BD Pharmingen) to label only activated astrocytes or PE-conjugated anti-mouse CD45 (1:200, 30F11, eBioscience) in TBST to label general immune cells. After incubation, excess stain was removed and sections were washed thrice with TBST for 10 min at room temperature, followed by a deionised water wash for 5 min. Sections were cover-slipped using DAPI ProLong Diamond Antifade Mountant (Invitrogen) to stain all nuclei and were left to cure for a minimum of 1 h at 4 °C in the dark. The 18-week cohort was stained with primary mouse anti-Iba1 (1:200, NCNP24, Wako Chemicals) and secondary anti-rabbit IgG (1:200, AlexaFluor-568, A-11011, Life Technologies) according to the manufacturer’s recommended instructions. To perform neuronal counts, the sections were stained with guinea pig anti-mouse NeuN (1:1000, MAB3777, Merck Millipore) and donkey anti-guinea pig IgG (1:400, AlexaFluor-647, Jackson Immunoresearch).

### Quantification of neurons and immune cells

Imaging of Immunofluorescence sections was performed on a Nikon C1 confocal laser-scanning microscope (Hamamatsu, Japan) at × 20 magnification with 405 nm (DAPI), 568 nm (PE) and 637 nm (APC) lasers. All microscope settings and laser powers were kept identical between each batch of sections imaged. Cell counts were performed manually, under blinded conditions, selected by appropriate cell body co-localisation of DAPI^+^ and either CD45^+^, IBA1^+^, or NeuN cells using FIJI (v1.51, NIH). Adjustments to brightness and contrast were made to individual files to allow optimal visualisation of CD45^+^, Iba1^+^ and NeuN^+^ cells only. Quantification of GFAP^+^ activated astrocytes per area stained was performed using identical threshold settings for all samples within each cohort using FIJI.

### Cytometric bead array

Plasma cytokine levels were measured by cytometric bead array (CBA) using the BD CBA Mouse Th1/Th2/Th17 Cytokine Kit (BD Biosciences, San Jose, CA, USA). The kit was used for parallel detection of mouse interleukin-2 (IL-2), interleukin-4 (IL-4), interleukin-6 (IL-6), interferon-γ (IFNγ), tumour necrosis factor (TNF), interleukin-17A (IL-17A) and interleukin-10 (IL-10). Standards were reconstituted with 2 mL of assay diluent to a maximum concentration of 5000 pg/mL and serially diluted to a concentration of 2.5 pg/mL. Capture bead mix was prepared by adding 60% of total capture beads to 40% assay diluent as per the kit instructions. Thereafter, 25 μL of bead mix, 25 μL of plasma and 15 μL phycoerythrin (PE) detection reagent were added consecutively to each well and incubated at room temperature for 2 h in the dark. Samples were washed and resuspended in wash buffer. Assay diluent was used as an internal control in place of sample plasma and returned negative values for all cytokines. Samples were measured using the Navios flow cytometer (Beckman Coulter Inc) and analysed using FlowJo (v 10.3.0). Standard curves and interpolations were constructed using GraphPad Prism 7.0 software.

### Real-time in vivo imaging of cerebral vasculature

To examine the leukocyte-endothelial cell interactions in the brain at experimental endpoints, intravital multiphoton microscopy of the brain was performed. Mice were anaesthetised by IP injection of an anaesthetic cocktail consisting of 150 mg/kg ketamine hydrochloride and 10 mg/kg xylazine and the tail vein was cannulised to administer fluorescently labelled antibodies and/or additional anaesthetic, if required. Body temperature was maintained using a heat pad. Mice were immobilised in a stereotactic frame, whereby the skin overlying the skull was swabbed with ethanol and a 1 cm vertical incision was made to expose the skull. The skin was retracted, and a 5 mm × 5 mm cranial window was shaved to a thin membrane using a dental drill. PE-conjugated anti-mouse CD31 (390, 1 mg/25 g mouse, eBioscience) and PacBlue-conjugated anti-mouse GR1 (RB6-8C5, 1 mg/25 g mouse, eBioscience,) were injected through the intravenous cannula to label the cerebral brain vasculature and neutrophils/monocytes, respectively. At least three post-capillary venules of approximately 20–40 μm in diameter were chosen per mouse. Images and videos were acquired using a multiphoton microscope (Leica SP5), equipped with a × 20 water-dipping objective (NA 1.0) and a MaiTai pulsed infrared laser (SpectraPhysics) set to an excitation wavelength of 810 nm. A 512 × 512 pixel image was acquired every 1.5 s for 9 min. Adjustments to brightness and contrast were made to individual files to allow optimal visualisation and measurements of blood vessels and cells. Multiple measurements of the width of blood vessels were taken and averaged, while the length of the vessel was measured along its centre, both using the segmented line tool. The number of adherent, intravascular cells was normalised to the vessel surface area and time of recording. Cells were considered adherent if they interacted with the vessel wall for 30 s or more. The measurements from all fields of view from one mouse were averaged with the final data representing neutrophil/monocyte numbers from one mouse.

### Statistical analysis

All statistical analyses were performed using GraphPad Prism Software (La Jolla, CA, USA). The normality of all data were first assessed using a Shapiro-Wilk normality test and analysed using a one-way analysis of variance (ANOVA) or Kruskal-Wallis posthoc test if appropriate. Each group was compared to the CIT + CON group. Some data were excluded due to outliers, determined using Grubbs’ test (Prism 7) at *P* ≤ 0.05. All values are mean ± SEM. A value of *P* ≤ 0.05 was considered statistically significant.

## Results

### Establishment of high-fat feeding and hyperglycemia experimental models at 9-week time point

**Fig. 1:**
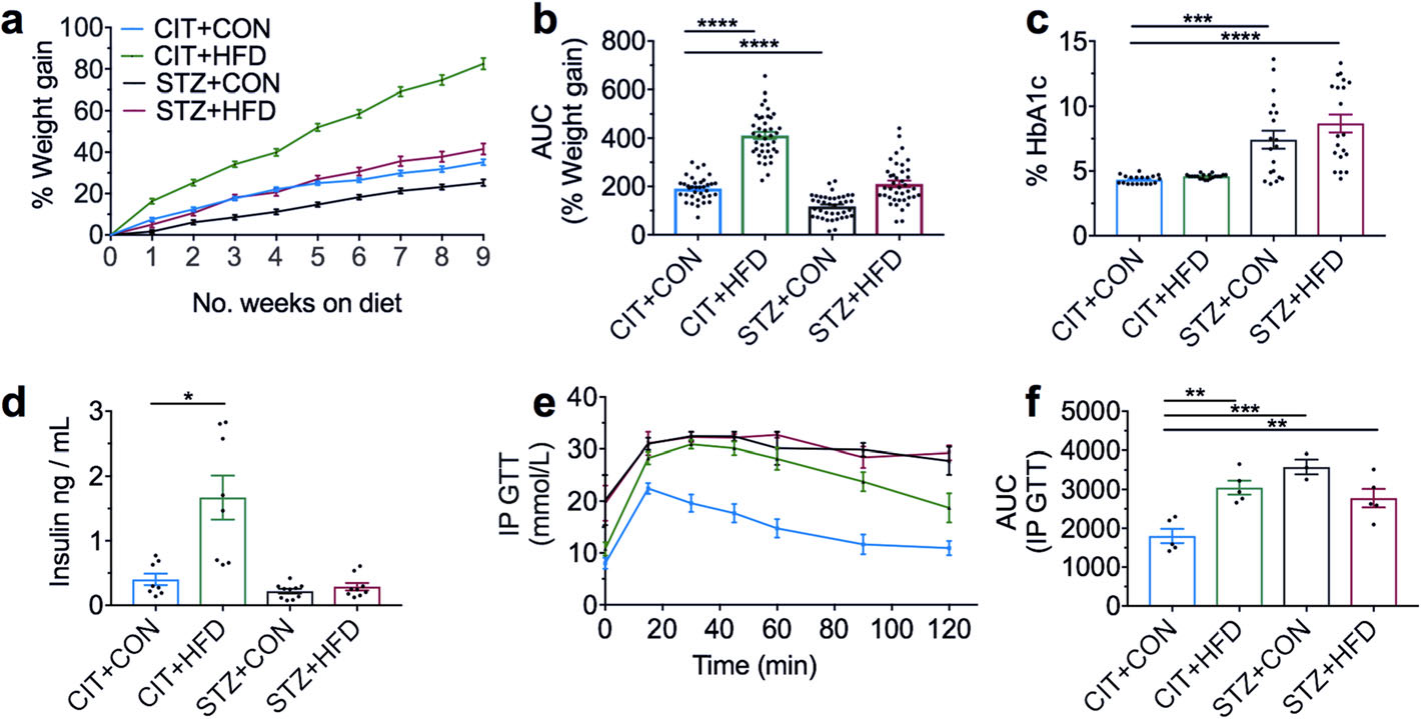
Diet-induced weight gain and STZ-induced hyperglycemia after 9 weeks. Male C57BL/6 J mice (5–6-week-old) were treated IP with either citrate vehicle (CIT) or streptozotocin (STZ; 55 mg/kg) 3, 5 and 7 days prior to commencement of a control diet (CON; 16% kilojoules from lipids) or high-fat diet (HFD; 44% kilojoules from lipids) for 9 weeks. **a** Mice were weighed every 3–4 days, and percentage change in body mass compared to baseline following the high-fat diet (HFD), control diet (CON) and STZ treatment was calculated at the end of each week. **b** Corresponding percentage weight gain as the area under the curve (AUC). **c** Percentage of glycated haemoglobin (HbA1c) from heparanised whole blood was measured as an indicator of hyperglycemia at the endpoint. **d** Plasma levels of insulin at the endpoint. **e** Following a 3 h fast, blood glucose measurements of the vehicle and STZ-induced mice were assessed via tail tip vein collection (time zero) and then administered 0.8 mg/g glucose solution for IP glucose tolerance test (GTT). Blood samples were collected from the tail tip vein at 15, 30, 45, 60, 90 and 120 min after glucose challenge. **f** Corresponding AUC of the IP GTT. Data are shown as mean ± S.E.M. of **a** and **b**, *n* = 39–40; **c**, *n* = 19–20; **d,** *n* = 8–10; **e** and **f**, *n* = 3–5 per group. Data in **c** and **d** analysed by Kruskal-Wallis test followed by Dunn’s, **b** and **f** by one-way ANOVA with Dunnett’s. *P < 0.05, **P < 0.01, ***P < 0.001, ****P < 0.0001 vs CIT + CON control group

To investigate the effect of high-fat feeding on neuroinflammation, we first established a mouse model with high-fat feeding. We show that mice on a HFD (CIT + HFD) exhibited significant weight gain compared to mice fed on a control diet (CIT + CON) as confirmed by the area under the curve (AUC) (Fig. 1a, b). In contrast, STZ-treated mice (STZ + CON) had retarded weight gain compared to CIT-treated mice (CIT + CON) on the same diet (Fig. 1b). Despite this, all STZ-treated mice developed hyperglycemia, regardless of diet, as determined by the increase in percentage glycated haemoglobin at the 9-week endpoint (Fig. 1c). However, high-fat feeding alone did not alter the percentage of glycated haemoglobin at the 9-week endpoint (Fig. 1c), likely attributable to significantly elevated plasma insulin in this group of mice (Fig. 1d).

Next, we performed a glucose tolerance test to assess glucose absorption from the bloodstream in our experimental groups. At the 9-week experimental endpoint, a 20% glucose solution was injected into fasting mice and blood glucose levels were assessed at various time points thereafter. As shown in the CIT + CON group, the metabolism of glucose is normally cleared from the bloodstream at 90 min post-injection (Fig. 1e). On the contrary, all of the other three experimental groups demonstrated marked delay in the absorption of circulating glucose, failing to return to baseline levels after 120 min (Fig. 1e). As confirmed by AUC, all three experimental groups demonstrated significant impairment in glucose tolerance when compared to mice in the CIT + CON group at the 9-week experimental endpoint (Fig. 1f). Taken together, we have established a high-fat feeding only group (CIT + HFD) whereby the mice are significantly overweight and demonstrate impairment in glucose tolerance. The mice in the STZ + CON group exhibit elevated blood glucose, increased percentage of glycated haemoglobin and inefficient glucose metabolism, forming the hyperglycemia-only group.

### High-fat feeding and hyperglycemia modify immune populations in the brain

**Fig. 2:**
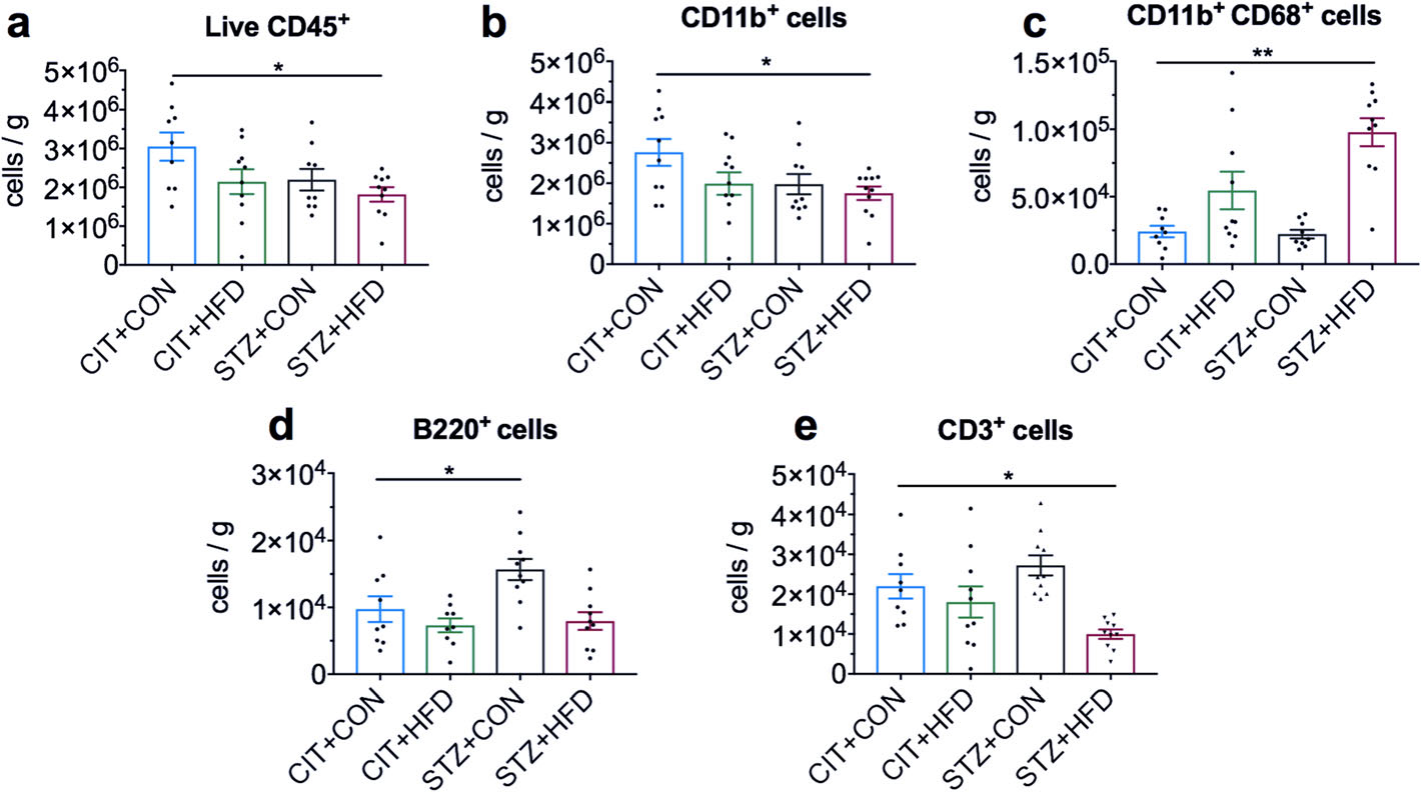
High-fat feeding and hyperglycemia contribute to global immune changes in the brain after 9 weeks. **a** Flow cytometry analysis of brain CD45^+^7AAD^−^ leukocytes of the left cerebral hemisphere. **b** Gated cells were analysed for the expression of CD11b as a marker of neutrophils, monocytes and microglia. **c** CD11b^+^ cell gate was further analysed for expression of CD68 as a similar marker. **d** Gated cells for B220 as a marker of B cells and (**e**) CD3 as a marker of T cells were analysed from the live CD45^+^ population. Data are shown as mean ± S.E.M. of **a**–**e,** *n* = 9–10 per group. Data in **a**, **b, d, e** analysed by one-way ANOVA with Dunnett’s. **c** Analysed by Kruskal-Wallis with Dunn’s. *P < 0.05, **P < 0.01 vs CIT + CON control group

To assess the effect of high-fat feeding, hyperglycemia, or a combination of both, on the immune changes in the brain at the 9-week experimental time point, we used flow cytometry to quantify innate and adaptive immune cells present in the cerebral tissue. Using CD45 as a pan-leukocyte marker, we identified approximately 3 × 10^6^ cells per gram of hemispheric tissue in the CIT + CON control group (Fig. 2a). Interestingly, mice treated with STZ on a high-fat diet exhibited significantly less CD45^+^ cells compared to the CIT + CON control group (Fig. 2a). Moreover, the majority of these CD45^+^ live cells were CD11b^+^ and were significantly decreased in STZ + HFD mice (Fig. 2b). Despite lower CD45^+^CD11b^+^ numbers, the number of CD11b^+^CD68^+^ cells was significantly increased in STZ + HFD mice despite reduced CD45^+^ leukocyte numbers (Fig. 2c). Using B220 and CD3 as general B and T cell markers respectively, we observed that mice in the hyperglycemia-only group (STZ + CON) showed a significant increase in the number of B cells (Fig. 2d), whilst those on high-fat diet (STZ + HFD) exhibited a significant decrease in the numbers of T cells compared to control (Fig. 2e). Collectively, these data indicate more robust changes in the cerebral immune population were seen in STZ-treated mice, particularly in the presence of a high-fat diet after 9 weeks.

### Hyperglycemia increases microglia numbers and activates astrocytes

**Fig. 3:**
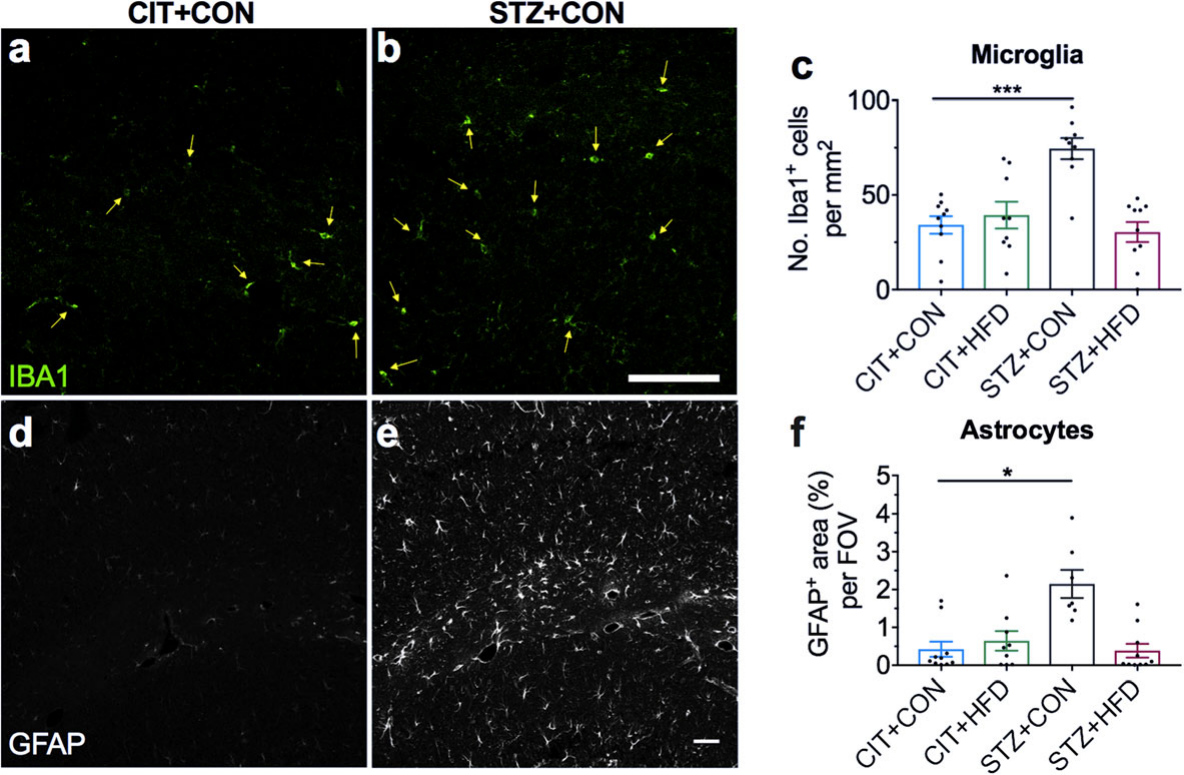
Hyperglycemia alone contributes to the hippocampal increase of microglia and astrocyte gliosis after 9 weeks. Representative coronal brain sections for immunofluorescence detection of Iba1^+^ microglia (green) and GFAP^+^ astrocytes (grey) in the vehicle (**a**, **d**) and STZ-treated (**b**, **e**) mice on control diet in CA1b of the hippocampus. Scale bars, 50 μm (**b**) and 100 μm (**e**). **c** Quantification of microglia per mm^2^. **f** Quantification of astrocytes as percentage area stained per FOV. Data represent mean ± S.E.M. of *n* = 8–10 per group. **c** One-way ANOVA with Dunnett’s. **f** Kruskal-Wallis with Dunn’s. *P < 0.05, ***P < 0.001 vs CIT + CON control group

The hippocampus is a region of the brain responsible for consolidating short and long-term memory and has been shown to be particularly prone to inflammation and damage during metabolic disease [15]. Therefore, we next examined whether there is evidence of neuroinflammation localised to CA1b of the hippocampus in our models of MetS. Using immunofluorescence, we quantified the numbers of Iba1^+^ microglia and assessed the area of astrocytosis as denoted with GFAP^+^ staining. At the 9-week experimental endpoint, mice in the STZ + CON group showed significantly increased numbers of microglia (Fig. 3a–c). In addition, the degree of astrocytosis in CA1b of the hippocampus was more pronounced in the STZ + CON group compared to CIT + CON control (Fig. 3d**–**f). Interestingly, no significant increases in microglia number were observed in any of the experimental groups in sections of the cortex (Additional file 1: Figure S1), suggesting a regional-specific effect. Intriguingly, the enhanced microglial numbers and astrocytosis were not observed in the CIT + HFD or STZ + HFD group (Additional file 1: Figure S2), suggesting that increased microglial numbers and astrocytosis as indicators of inflammation after hyperglycemia may be mitigated by the effects of a high-fat diet. To assess whether the hyperglycemia-induced gliosis was associated with neurodegeneration or neuronal loss, we quantified the number of NeuN^+^ cells in the pyramidal layer of CA1, its thickness and general NeuN^+^ cells in CA1 (Additional file 1: Figure S3), but noted no significant change.

### Establishment of chronic high-fat diet and hyperglycemia experimental models at 18-week time point

**Fig. 4:**
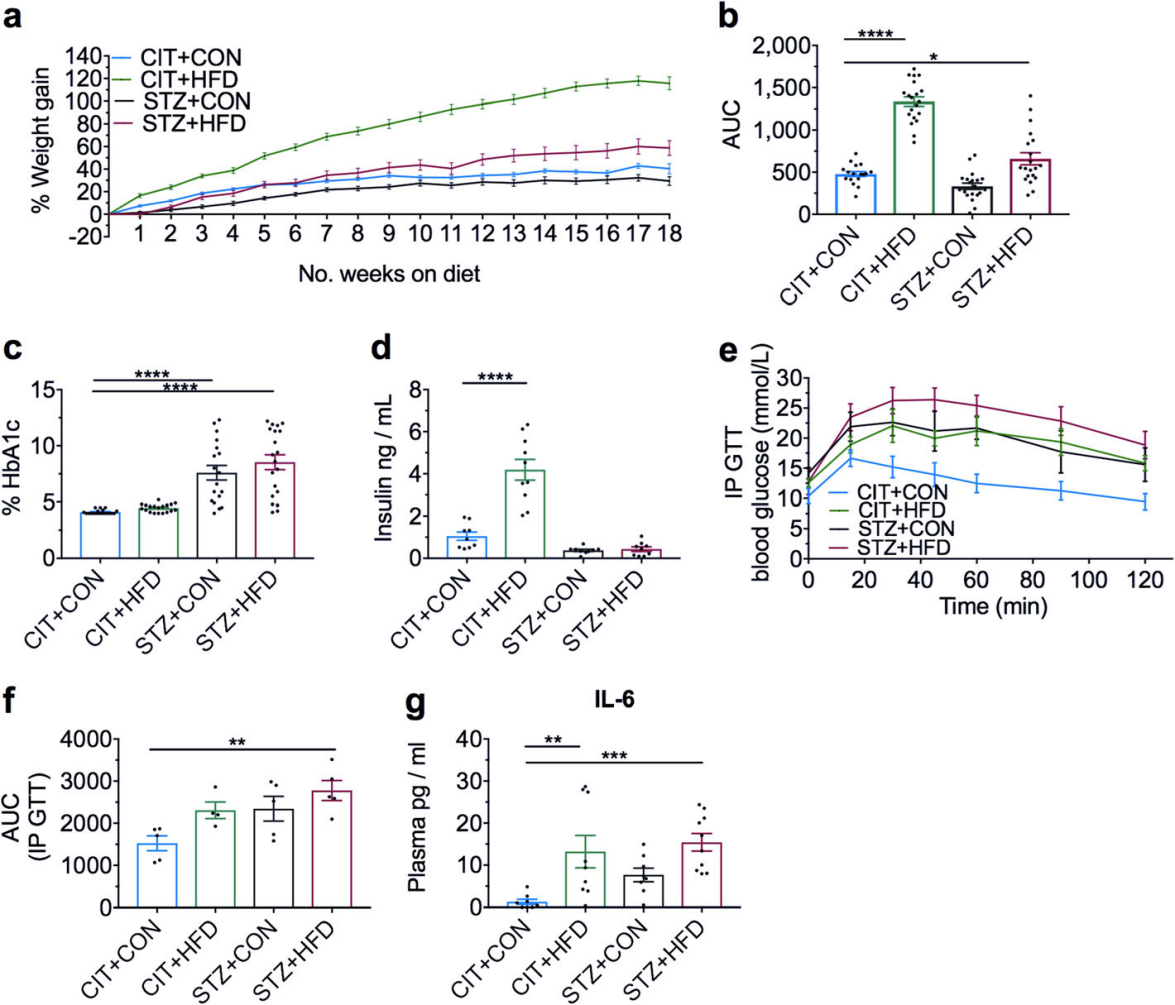
Diet-induced weight gain and STZ-induced hyperglycemia after 18 weeks. **a** Mice were weighed every 3–4 days and percentage gain in body mass compared to baseline following the high-fat diet (HFD), control diet (CON) and STZ treatment was calculated at the end of each week. **b** Corresponding percentage weight gain as the area under the curve (AUC). **c** Percentage of glycated haemoglobin or HbA1c from heparanised whole blood was measured as an indicator of hyperglycemia at the endpoint. **d** Plasma levels of insulin from whole blood. **e** Following a 3 h fast, blood glucose measurements of the vehicle and STZ-induced mice were assessed via tail tip vein collection (time zero) and then administered 0.8 mg/g glucose solution for IP glucose tolerance test (GTT). Blood samples were collected from the tail tip vein at 15, 30, 45, 60, 90 and 120 min after glucose challenge. **f** Corresponding AUC of the IP GTT. **g** Proinflammatory cytokine interleukin-6 was measured from plasma using cytometric bead array. Data are shown as mean ± S.E.M. of **a**–**c**, *n* = 18–20; **d,** *n* = 9–10; **e** and **f**, *n* = 4–5; **g**, *n =* 8–10 per group. **b**, **d** and **f** analysed by ordinary one-way ANOVA with Dunn’s. **c** and **g** analysed by Kruskal-Wallis with Dunnett’s. *P < 0.05, **P < 0.01, ***P < 0.001, ****P < 0.0001 vs CIT + CON control group

To examine whether the brain immune population changes and inflammatory state worsened with a longer period of a high-fat feeding and/or hyperglycemia, we assessed a separate cohort of animals on these experimental parameters after 18 weeks. Both CIT- and STZ-treated mice fed a HFD exhibited significant weight gain over 18 weeks compared to CIT + CON group, gaining up to 110% and 50% of their original body weight respectively (Fig. 4a, b). Similar to the findings of the 9-week model, all STZ-treated mice developed hyperglycemia as determined by the increase in percentage of glycated haemoglobin at the 18-week endpoint (Fig. 4c). However, high-fat feeding on its own (CIT + HFD) did not elevate the percentage of glycated haemoglobin at the 18-week endpoint (Fig. 4c). Again, this may be attributable to the substantially elevated plasma insulin in this group of mice (Fig. 4d).

In addition, we performed glucose tolerance testing to assess any impairment in glucose absorption from the bloodstream that could suggest the presence of insulin resistance in the experimental groups after 18 weeks. As shown in our CIT + CON group, the metabolism of glucose is normally cleared from the bloodstream at 90 min post-injection (Fig. 4e). In contrast to the findings of the 9 weeks experimental endpoint, only STZ-treated mice on high-fat diet (STZ + HFD) demonstrated a significant impairment in glucose tolerance compared to CIT + CON controls at 18 weeks after the onset of the model. This was confirmed by the quantification of the AUC (Fig. 4f). Furthermore, as both chronic high-fat feeding and hyperglycemia are associated with systemic inflammation both clinically and experimentally, we assessed systemic levels of pro-inflammatory IL-6 in our respective models. Both CIT- and STZ-treated mice on a HFD exhibited significantly greater levels of circulating IL-6 compared to CIT + CON controls. (Fig. 4g). No detectable levels of cytokines were found in the 9-week cohort (data not shown).

### The effect of chronic high-fat feeding and hyperglycemia on the brain

**Fig. 5:**
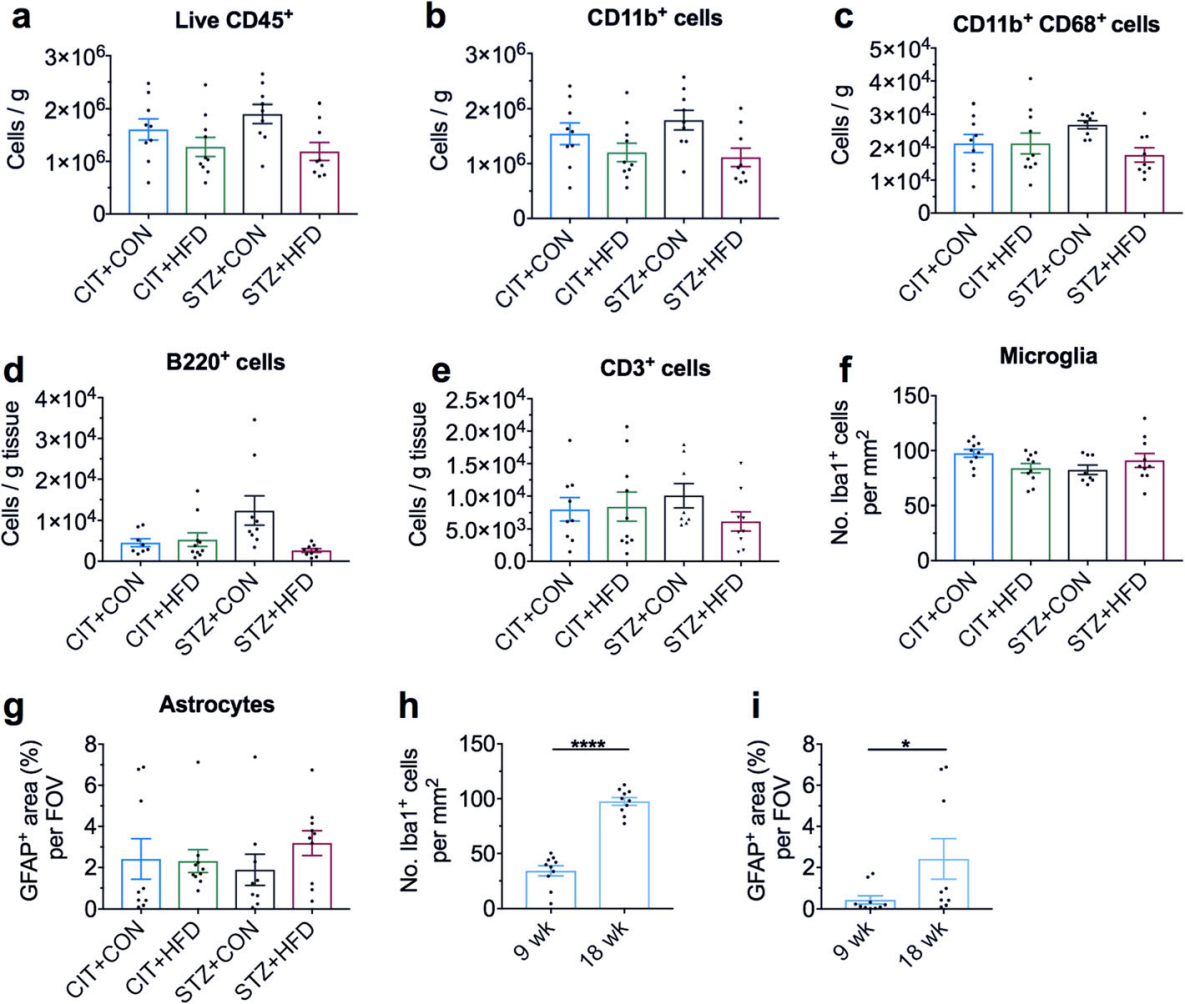
Global brain immune changes return to baseline after 18 weeks of high-fat feeding and/or hyperglycemia. **a** Flow cytometry analysis of brain CD45^+^7AAD^−^ leukocytes of the left cerebral hemisphere. **b** Gated cells were analysed for the expression of CD11b as a marker of neutrophils, monocytes and microglia. **c** CD11b^+^ cell gate was further analysed for expression of CD68 as a phagocytic marker. **d** Gated cells for B220 as a marker of B cells and (**e**) CD3 as a marker of T cells were analysed. **f** Number of Iba1^+^ microglia and (**g**) GFAP^+^ astrocytes in CA1 of the hippocampus were quantified at 20 weeks, and (**h**) microglial and (**i**) astrocyte numbers were compared between time points in CIT + CON mice. Data are shown as mean ± S.E.M. of **a**–**e,** *n* = 8–10 per group. Data in **a–c** analysed by one-way ANOVA with Dunnett’s. **d** and **e** analysed by Kruskal-Wallis with Dunn’s. **h** and **i** by Student’s *t* test. *P < 0.05, ****P < 0.0001 vs CIT + CON control group

We provided support for the presence of neuroinflammation (altered immune populations, increased number of hippocampal microglia and astrocytosis) mostly in the STZ + CON group at the 9-week time point. We next assessed these parameters in the long-term model after 18 weeks of treatment. We hypothesised that the elevated systemic inflammation evident at the 18-week time point would elicit more pronounced neuroinflammation in the brain compared to 9 weeks of high-fat feeding and hyperglycemia. Interestingly, we found no significant differences in the numbers of total leukocytes, CD11b^+^ and phagocyte-like cells (Fig. 5a–c), or in the number of B (Fig. 5d) and T cells (Fig. 5e) in the brains of any of the experimental groups compared to the CIT + CON control counterparts. However, it is important to note that the number of the various immune populations reported here in the chronic model was distinctly different to the findings of the 9-week time point. In fact, the total number of leukocytes per gram of brain tissue was approximately 50% less in the 18-week experimental groups compared to 9-week, and this was consistent for both innate and adaptive immune populations. Similarly, despite the lack of significant changes in the number of microglia (Fig. 5f) or degree of astrocytosis (Fig. 5g) in the hippocampus of any of the experimental groups compared to the CIT + CON controls at the 18-week time point, the number of Iba1^+^ microglia and the degree of activated astrocytes denoted by GFAP^+^ staining were elevated in an age-dependent manner. Indeed, there was a ~ 5-fold and 2.5-fold increase in Iba1^+^ cells and GFAP^+^ staining in CIT + CON mice at 18 weeks compared to the 9-week cohort (Fig. 5h, i), suggesting ageing supersedes high-fat or hyperglycemia-induced inflammation on the brain.

### Chronic hyperglycemia promotes cerebral leukocyte-endothelial cell interactions

**Fig. 6:**
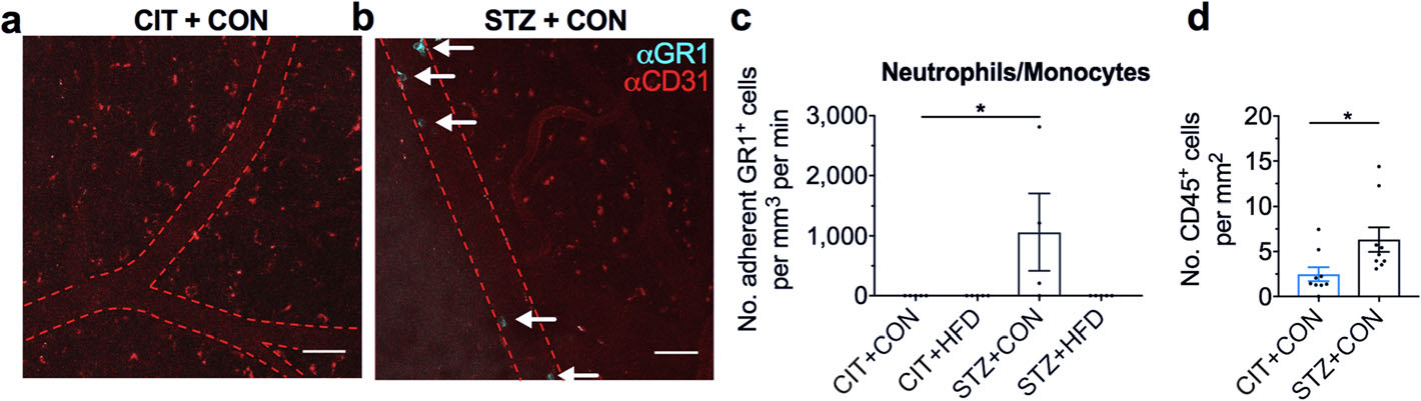
Hyperglycemia drives peripheral recruitment of neutrophils and monocytes to the brain after 18 weeks. **a** Intravital multiphoton microscopy of the cerebral vasculature of vehicle-treated mice on control diet. **b** Intravital multiphoton microscopy of the cerebral vasculature of STZ-treated mice on control diet. Monocytes/neutrophils are visualised in blue, labelled with anti-GR1 mAb (clone RB6-8C5). Endothelium is visualised in red, labelled with anti-CD31 mAb and outlined with the red dotted line. Scale bars, 50 μm. **c** Quantification of GR1+ neutrophils/monocytes. Each data point represents the average of three fields of view per mouse. **d** Immunofluorescence of CD45^+^ leukocytes in the cortex. Data represent mean ± S.E.M. of *n* = 4–5 per group. Kruskal-Wallis with Dunn’s. *P < 0.05 vs CIT + CON control group

To assess whether the regional-specific neuroinflammation observed in the hyperglycemic mice at 9 weeks or systemic inflammation evident in the mice on 18 weeks of HFD was associated with recruitment of peripheral immune cells to the brain, we used intravital multiphoton microscopy to assess cerebral leukocyte-endothelial interactions. We delivered fluorescently conjugated anti-GR1 antibody into mice intravenously to study the interaction of monocytes and/or neutrophils with the cerebral vasculature in vivo. We did not observe any cerebral leukocyte-endothelial cell interactions in all groups at the 9-week time point (data not shown). However, after 18 weeks following induction of hyperglycemia, there was a significant increase in the number of GR1^+^ leukocytes adherent to the post-capillary venules in the brain of mice from the STZ + CON group (Fig. 6a–c). No cerebral leukocyte-endothelial cell interactions were detected in any other experimental groups. In addition, to examine if chronic hyperglycemia induces the recruitment of blood-derived leukocytes into the brain, we used immunofluorescence to quantify the numbers of CD45^+^ cells in the cortex of a separate cohort of mice. We found a significantly increased number of CD45^+^ cells in the cortex of STZ + CON mice compared to control (Fig. 6d).

## Discussion

In this study, we dissect the specific contributions of high-fat feeding and hyperglycemia to neuroinflammation in experimental mouse models of MetS. We demonstrate that acute hyperglycemia induces regional-specific effects on the brain by elevating microglial numbers and promotes astrocytosis in the hippocampus. In addition, we demonstrate that chronic hyperglycemia supports the recruitment of peripheral leukocytes, particularly GR1^+^ granulocytes, to the cerebral microvasculature. Moreover, we provide evidence that these changes are independent of the systemic inflammation associated with high-fat feeding. Taken together, our findings suggest that hyperglycemia rather than high-fat feeding contributes to the development of regional neuroinflammation in animal models of MetS.

MetS in humans is associated with a wide-array of phenotypes. Therefore, the need for clinically relevant and highly reproducible animal models is paramount. Research has primarily centered on using monogenetic deletions or mutations that indirectly induce severe obesity, yet in the case of T2D, obesity is usually second only to environmental influences, not manifestations of genetic mutations [16]. Thus, the clinical relevance of these models to obesity in MetS and T2D in humans remains controversial. In this study, we used a high-fat diet as a method of inducing weight gain and low-dose STZ-treatment in producing a form of mild hyperglycemia. While a definitive diagnosis of hyperglycemia in T2D can vary depending on the weight, gender and race of an individual, clinical benchmarks constitute mild hyperglycemia as > 7–9% glycosylated haemoglobin [17]. Our findings indicate that the STZ treatment regime induced a clinically comparable mild hyperglycemia (8–9%), which was sustained over both 9-week and 18-week time points. To induce weight gain, high-fat feeding was selected to reflect the macronutrient composition of the westernised diet, strongly implicated in the development of obesity and insulin resistance in MetS [18]. We found that high-fat feeding (ad libitum) significantly induced weight gain. Similarly, high-fat feeding was associated with significant impairments to glucose metabolism and hyperinsulinaemia, but not hyperglycemia. Previous studies of HFD in C57Bl/6 J mice note percentage weight gain and insulin resistance consistent with our findings, yet contrastingly, with induced hyperglycemia, which may in part be due to the composition of lipids in our diet (22% fat), compared to these studies (60% fat) [19]. Our attempt of a combination model (STZ + HFD) resulted in weight gain at 18 weeks, but not 9 weeks. The delayed progression of weight gain in the mice of STZ + HFD group may be attributed to preferential lipid metabolism following impairment of glucose uptake. While only observational, these are critically important findings. Our use of high-fat feeding and STZ-treatment seemingly echo a sounder model of weight gain and hyperglycemia after 18 weeks, reflecting similar conditions and levels relevant to those seen in clinical MetS, without the need of gene deletions or mutations to induce these conditions. Although beyond the scope of this study, it would be important to validate whether our models demonstrate the diverse comorbidities of MetS, such as cardiovascular disease, nephropathy and atherosclerosis. The presence of these complications that arise from MetS patients would lend credence to our models in more accurately representing the more complex physiology and clinical presentation of MetS patients.

Administration of STZ alone to male C57BL/6 mice is commonly associated with retarded weight gain, as we and others routinely observe [20]. Similar retarded weight gain is also evident in rats subjected to STZ alone [21], as well as other mouse models where there is also hyperglycemia in the absence of high-fat diet [22, 23]. This retarded weight gain is a direct result of the degree of hyperglycemia, as it is not evident at a milder hyperglycemia [21], and is preventable by insulin replacement [23]. Rather than proposing that high glucose protects against the weight-gaining effect of a HFD, we would hence propose that a HFD protects against the retarded weight gain effect of hyperglycemia in rodent models. This protective effect of HFD on retarded weight gain as a result of impaired insulin availability may reflect a specific rodent phenomenon rather than a potential means of preventing obesity in diabetic humans. Interestingly, however, patients diagnosed with type 1 diabetes (insulin deficiency) exhibit a more severe hyperglycemia than patients diagnosed with type 2 diabetes (insulin resistance), yet it is the latter that are more commonly associated with obesity [24]. Clinical and experimental evidence has established that chronic hyperglycemia and weight gain in MetS are associated with both localised and systemic inflammation, yet studies struggle to delineate this from the effects of hyperglycemia alone. Our current study is able to shed light on this. By giving a HFD to both CIT- and STZ-treated mice, we examined whether systemic inflammation was driven solely by high-fat feeding, or in conjunction with hyperglycemia. Our findings show that mice in the CIT + HFD and STZ + HFD groups had increased levels of systemic pro-inflammatory IL-6 after 18 weeks, but not STZ + CON mice, suggesting that high-fat feeding is more likely to contribute to systemic inflammation over hyperglycemia. Previous experimental studies of high-fat feeding in C57Bl/6 J mice note significant increases in IL-6 and other pro-inflammatory cytokines after only 8 weeks [25], while studies involving the use of STZ-treatment in mice vary, noting the presence of IL-1β and TNFα in pancreatic tissue [26] and IFNy and TNFα systemically [27]. In accordance with these studies, our results demonstrate that chronic weight gain induced by 18 weeks of high-fat feeding impacts significantly on systemic inflammation.

Inflammation is recognised as a key mechanism of diabetes progression and its multifactorial complications; its likely contribution to diabetes-induced neuropathy is now also clearly emerging [28]. Indeed, diabetes is regarded as a low-grade, chronic inflammatory disorder [29, 30]. Systemic inflammation is unmistakably present in diabetic patients [31, 32] and is considered a contributing mechanism to peripheral disease progression [33]. CD68 is a protein highly expressed by monocytes and macrophages, as well as microglia. Our observation that a combination of hyperglycemia and high-fat feeding leads to elevated CD11b^+^CD68^+^ cells is consistent with a diabetes-driven phenotype of increased circulating monocytes and tissue microglia, and suggests that inflammatory signalling is also likely to be implicated in both the systemic and central complications of diabetes, analogous to the known contribution of inflammation to other neurological disorders [28].

Under steady-state conditions, the number and function of microglia are tightly regulated, comprising the immune-dominant cell type in the brain. In response to an acute immune stimulus, microglia proliferate, increase phagocytosis, and clear the pathogenic insult—a process referred to as priming [34]. Generally, microglia will undergo apoptosis upon resolving inflammation, but in response to a chronic stimulus, microglia may become notoriously long-lived, contributing to—and thus enhancing—neuro-destructive effects. In our study, mice in the STZ + CON group demonstrated marked increases in the number of hippocampal Iba1^+^ microglia after 9 weeks of treatment. This finding is consistent with a previous study that reported hippocampal-specific elevations in Iba1^+^ microglia in STZ-treated rats [35]. Moreover, mice in the STZ + CON group demonstrated a significant increase in activated astrocytes, as indicated by elevated GFAP staining. Intriguingly, these changes were seemingly ameliorated at the 18 weeks endpoint, with no difference found amongst the experimental groups. As neuroinflammatory responses are concomitant with the correct homeostasis functioning of the CNS and are capable of resolving over time, our findings suggest that the STZ-induced changes in microglial numbers and astrocytosis are representative of an acute insult, in this case, hyperglycemia. Chronic hyperglycemia has been associated with cerebral endothelial cell dysfunction and apoptosis, which may provide an entry-point for circulating inflammatory cytokines [36]. We propose that with more severe weight gain (i.e. obesity) and hyperglycemia, beyond the endpoints examined here, the effects of systemic inflammation and peripheral leukocyte recruitment could establish a form of chronic, non-resolving neuroinflammation in the brain. This would reflect the reasoning by which systemic cytokine increases in obese T2D patients are thought to become evident later in life and support the concept that neurological impairments are primarily seen in aged individuals [37]. While only conjecture, the notion that hyperglycemia could act as the primary insult in priming microglia prior to the systemic inflammation formed by the secondary insult (obesity and/or ageing) is an interesting prospect that remains to be fully examined.

Having established the presence of systemic inflammation in HFD-fed mice, we characterised whether this was associated with the presence of adherent leukocytes to the cerebral vasculature. The extravasation of leukocytes from the peripheral vasculature into the brain parenchyma is a critical component in both acute and chronic neuroinflammatory disorders. This phenomenon has been predominantly explored in rodent models of multiple sclerosis (MS) and ischaemic stroke in vivo [38, 39], yet the influences by which this occurs are not fully understood. Here, we observed adherent GR1^+^ granulocytes (neutrophils/monocytes) in the surface cerebrovasculature of STZ + CON mice after 18, but not 9 weeks. Interestingly, this adherence was not associated with any increases of IL-6 or other cytokines measured, suggesting that the recruitment of these cells is independent of systemic inflammation and high-fat feeding. Hyperglycemic conditions have been shown to induce endothelial cell dysfunction and promote low-grade inflammation [40]. Indeed, previous in vitro studies of human brain endothelial cell cultures reveal a high degree of apoptosis under hyperglycemic conditions [41]. To our knowledge, this is the first study of its kind to demonstrate in vivo granulocyte trafficking along the cerebrovasculature of hyperglycemic mice. Moreover, immunofluorescence of the cortex of STZ + CON mice after 18 weeks demonstrated an increase in the presence of CD45^+^ blood-derived leukocytes. Given the lack of changes in inflammatory cells and markers measured using flow cytometry at the level of hemispheric tissue, it is possible that the GR1^+^ granulocytes detected with intravital microscopy eventually transmigrated into the brain parenchyma and presented as this CD45^+^ population.

Although not specifically examined in this study, the notion of whether high-fat feeding under hyperglycemic conditions seemingly protected against the gliosis we observed at 9 weeks is an interesting prospect. High-fat feeding or the “ketogenic diet” has recently been shown to provide disease-modifying activity in a broad range of neurodegenerative disorders including Alzheimer’s disease [42]. These observations are supported by studies in animal models and isolated cells that showed ketone bodies, especially β-hydroxybutyrate, to confer neuroprotection against diverse types of cellular injury [43]. A recent study in young mice highlights that ketogenic diet intervention in mice induces significant increases in cerebral blood flow and P-glycoprotein transports on the blood-brain barrier to facilitate clearance of amyloid-β, a hallmark of Alzheimer’s disease [44]. Therefore, the absence of hyperglycemia-associated elevation of microglia and astrocytosis in the STZ + HFD group may further suggest a neuroprotective role of high-fat feeding.

Despite the overwhelming evidence linking ageing to a wide array of systemic and neuroinflammatory disorders [45], it is important to note that the mice used in these experiments were not considered aged. It would be pertinent, therefore, to repeat this investigation in aged mice to examine whether age-associated risk factors could provide the necessary threshold to drive chronic neuroinflammation. In support of this, we observed that both microglial numbers and the degree of astrocytosis in control mice hippocampus increased significantly by four and fivefold, respectively, between 9 and 18 weeks of treatment. Previous studies in aged mice also note increases in CA1-specific gliosis [46] and that both microglia and astrocytes are more susceptible to inflammatory stimuli [47]. However, impairments to microglial function have also been observed in aged mice with regard to phagocytosis and motility [48, 49], which may suggest that this increase in microglial numbers may be a mechanism of compensation for otherwise dysfunctional microglia. Indeed, this age-associated increase in key immune populations of the hippocampus may explain the increased vulnerability to cognitive functions of psychomotor speed and working memory in MetS patients [50].

## Conclusions

In summary, the findings of this study indicate that the hyperglycemic component of metabolic syndrome confers a more inflammatory role in the brain than high-fat feeding. Hyperglycemia alone promotes microglial numbers and astrocytosis in the hippocampus and is associated with the peripheral recruitment of leukocytes to the cerebrovasculature. On the contrary, chronic high-fat feeding is sufficient to induce a systemic inflammation, which may have other consequences on the brain over time. Further studies in older (12–18 months) mice will more accurately address the contributions of these components to the development of neuroinflammation and cognitive decline or dementia. Nevertheless, our results shed light on the specific contributions of high-fat feeding and hyperglycemia in the development of neuroinflammation.

## Additional file

Additional file 1: **Figure S1.** Microglial numbers in cortex was unchanged after 9 weeks. Cell counts of Iba1^+^ microglia in the cortex. Data represent mean ± S.E.M. of *n* = 8–10 per group. **Figure S2.** Representative coronal brain sections for immunofluorescence detection of Iba1^+^ microglia (green, denoted by yellow arrow) in the hippocampus of (a) CIT+HFD and (b) STZ+HFD mice. Scale bar, 100 μm. **Figure S3.** Neuronal numbers of CA1 hippocampus unchanged after 9-weeks of treatment. (a) Schematic diagram of the designated quantification area for neuronal counts. (b) Number of neurons in the pyramidal layer of CA1. (c) Area of pyramidal layer of CA1 (d) Number of neurons within CA1. Data represent mean ± S.E.M. of *n* = 8–10 per group. (DOCX 1055 kb)

## Abbreviations

AUC: Area under the curve
BMI: Body mass index
CA1: Cornu Ammonis 1
CD: Cluster of differentiation
CIT: Citrate vehicle
CON: Control diet
CVD: Cardiovascular disease
ELISA: Enzyme-linked immunosorbent assay
GFAP: Glial fibrillary acidic protein
GR1: Granulocytic marker 1
HFD: High-fat diet
IBA1: Ionised calcium-binding adapter molecule 1
IL-6: Interleukin 6
MetS: Metabolic syndrome
PFA: Paraformaldehyde
STZ: Streptozotocin
T2D: Type 2 diabetes
TNFα: Tumour necrosis factor alpha
