# Supplementary material for: Distinct contributions of hyperglycemia and high-fat feeding in metabolic syndrome-induced neuroinflammation
Source: J Neuroinflammation. 2018 Oct 22;15:293. doi: 10.1186/s12974-018-1329-8 (PMC6198529; doi:10.1186/s12974-018-1329-8)
Supplement: Supplementary file 1 — Figure S1. Microglial numbers in cortex was unchanged after 9 weeks. Cell counts of Iba1+ microglia in the cortex. Data represent mean ± S.E.M. of n = 8–10 per group. Figure S2. Representative coronal brain sections for immunofluorescence detection of Iba1+ microglia (green, denoted by yellow arrow) in the hippocampus of (a) CIT+HFD and (b) STZ+HFD mice. Scale bar, 100 μm. Figure S3. Neuronal numbers of CA1 hippocampus unchanged after 9-weeks of treatment. (a) Schematic diagram of the designated quantification area for neuronal counts. (b) Number of neurons in the pyramidal layer of CA1. (c) Area of pyramidal layer of CA1 (d) Number of neurons within CA1. Data represent mean ± S.E.M. of n = 8–10 per group. (DOCX 1055 kb) [file 12974_2018_1329_MOESM1_ESM.docx]

**Distinct contributions of hyperglycemia and high-fat feeding in metabolic syndrome-induced neuroinflammation**

Brooke J. Wanrooy^1^, Kathryn Prame Kumar^1^, Shu Wen Wen^1^, Cheng Xue Qin^2^, Rebecca Ritchie^2, 3^, and Connie H. Y. Wong^1,^ *

^1^ Centre for Inflammatory Diseases, Department of Medicine, School of Clinical Sciences at Monash Health, Monash Medical Centre, Monash University, Clayton, Victoria, Australia;

^2^ Baker Heart and Diabetes Institute, Melbourne, Australia;

^3^ Department of Diabetes, Monash University, Melbourne, Australia

*Address for correspondence: Connie H. Y. Wong, PhD., Centre for Inflammatory Diseases, Department of Medicine, School of Clinical Sciences at Monash Health, Monash Medical Centre, Monash University, Clayton, VIC 3168 Australia; Email: [connie.wong@monash.edu](mailto:connie.wong@monash.edu)


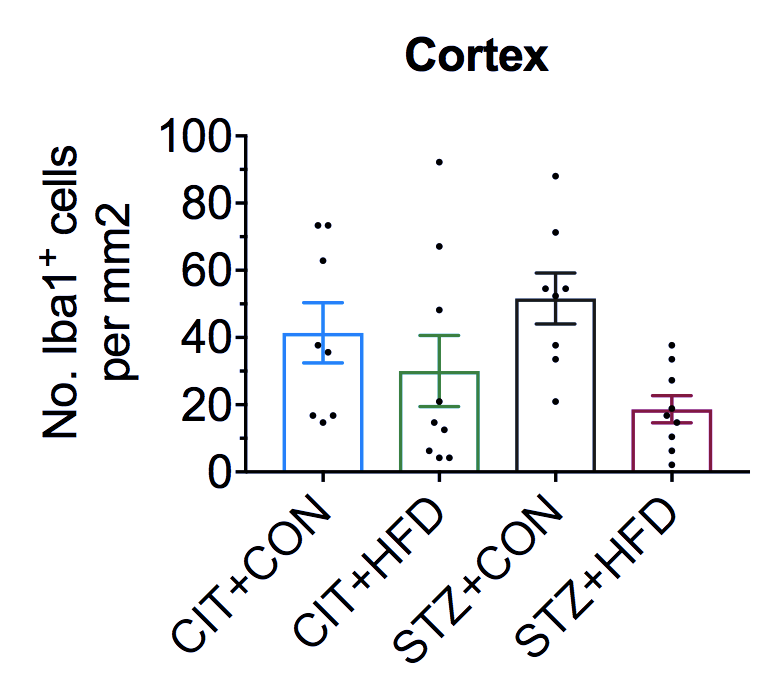


Figure S1: Microglial numbers in cortex was unchanged after 9 weeks. Cell counts of Iba1^+^ microglia in the cortex. Data represent mean ± S.E.M. of *n* = 8-10 per group.


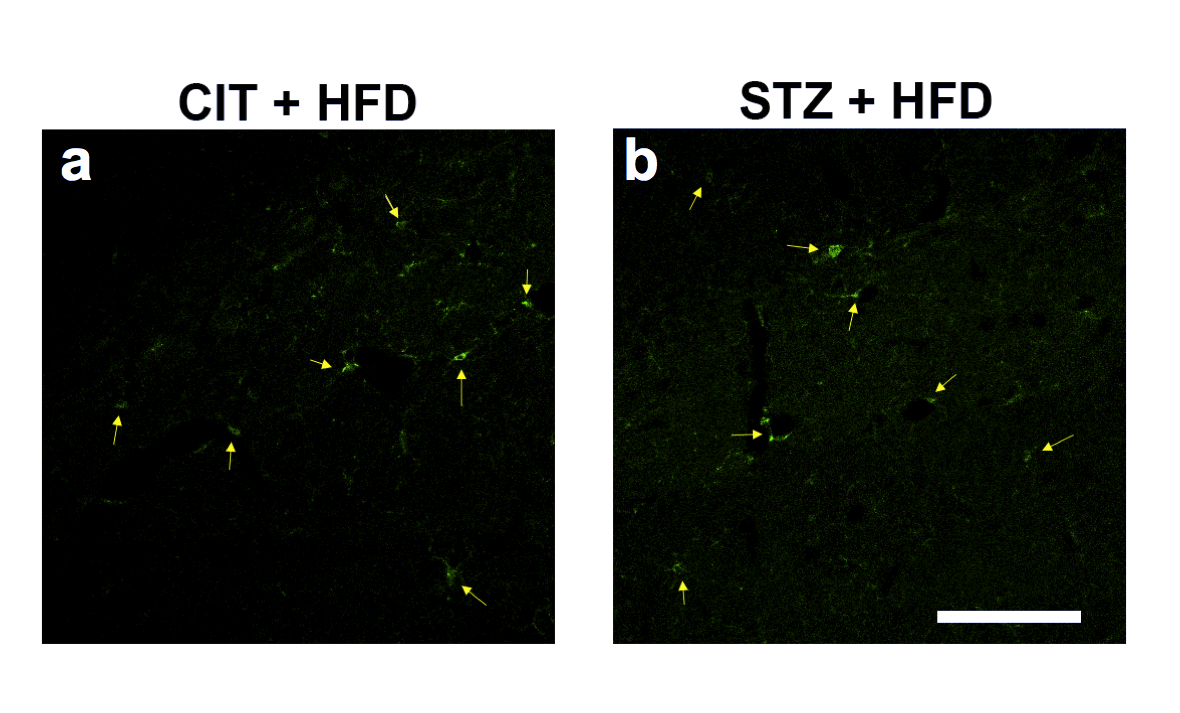


**Figure S2:** Representative coronal brain sections for immunofluorescence detection of Iba1^+^ microglia (green, denoted by yellow arrow) in the hippocampus of (a) CIT+HFD and (b) STZ+HFD mice. Scale bar, 100 μm.


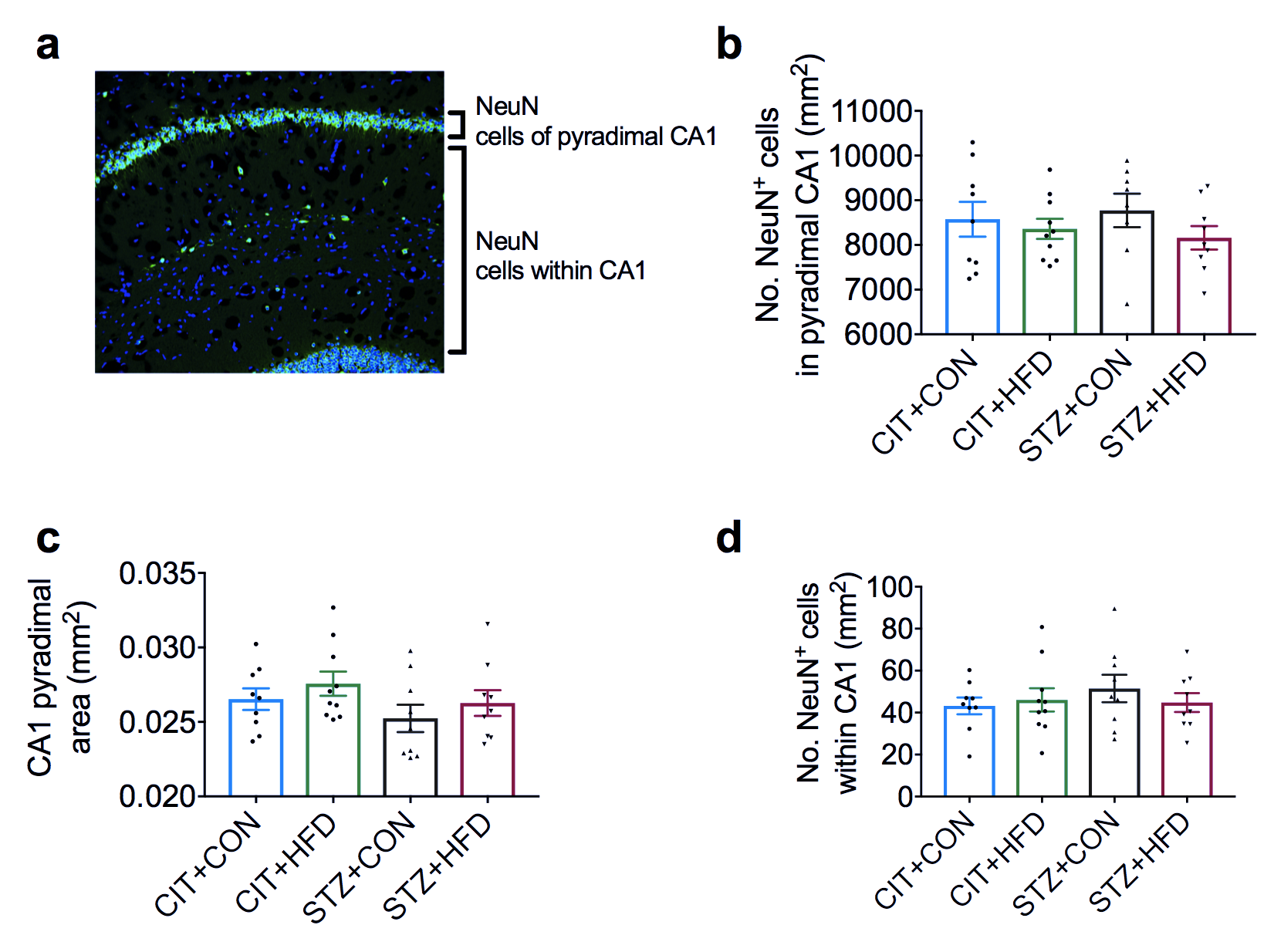


Figure S3: Neuronal numbers of CA1 hippocampus unchanged after 9-weeks of treatment. (a) Schematic diagram of the designated quantification area for neuronal counts. (b) Number of neurons in the pyramidal layer of CA1. (c) Area of pyramidal layer of CA1 (d) Number of neurons within CA1. Data represent mean ± S.E.M. of n = 8-10 per group.
